# Adipokines and Hepatic Insulin Resistance

**DOI:** 10.1155/2013/170532

**Published:** 2013-05-16

**Authors:** Yu Li, Lin Ding, Waseem Hassan, Daoud Abdelkader, Jing Shang

**Affiliations:** ^1^National Center for Drug Screening and State Key Laboratory of Natural Medicines, China Pharmaceutical University, Jiangsu Province 210009, China; ^2^Department of Pharmacy, Bahauddin Zakariya University, Multan 60800, Pakistan

## Abstract

Obesity is a major risk factor for insulin resistance and type 2 diabetes. Adipose tissue is now considered to be an active endocrine organ that secretes various adipokines such as adiponectin, leptin, resistin, tumour necrosis factor-*α*, and interleukin-6. Recent studies have shown that these factors might provide a molecular link between increased adiposity and impaired insulin sensitivity. Since hepatic insulin resistance plays the key role in the whole body insulin resistance, clarification of the regulatory processes about hepatic insulin resistance by adipokines in rodents and human would seem essential in order to understand the mechanism of type 2 diabetes and for developing novel therapeutic strategies to treat it.

## 1. Introduction

In many developed and developing countries, obesity has reached epidemic proportions, resulting in an increasing prevalence of type 2 diabetes characterized by insulin resistance of peripheral tissues such as liver, muscle, and fat which cannot be overcome by hypersecretion of pancreatic beta cells [1]. One survey conducted in 2000 revealed that more than 150 million people in the world suffered from type 2 diabetes [2] and 80% of these cases were related to obesity. Because various studies have demonstrated that hepatic insulin resistance plays a central role in the development of type 2 diabetes and obesity is centrally involved in increasing the clinical risk of diabetes, visceral adipose tissue is now thought to provide a link between obesity and hepatic insulin resistance.

Adipose tissue was traditionally regarded as a passive energy reservoir. However, since the discovery of leptin and subsequent identification of other adipose tissue-derived cytokines (e.g., adiponectin and resistin) in the last two decades [3], it became clear that adipose tissue is an active endocrine organ. Obese adipose tissue also secretes various inflammatory cytokines, such as interleukin-6 (IL-6) and tumour necrosis factor-*α* (TNF-*α*) [4]. All of these cytokines, termed adipokines, act in an autocrine, paracrine, or endocrine fashion to control various metabolic functions. Some of these adipokines have been implicated in the development of hepatic insulin resistance. Indeed, they may act locally or distally to alter insulin sensitivity in insulin-targeted organs such as liver which is also discussed in detail by Marra and Bertolani [5] previously or may act through neuroendocrine, autonomic, or immune pathways. For example, activation of proinflammatory pathways in adipose tissue is known to interfere with insulin signaling and induce hepatic insulin resistance [6]. Although, the role of adipocytokines on insulin resistance has been comprehensively elucidated elsewhere [7, 8], but recently specialized review on their actions in hepatic insulin resistance is missing. In this paper, we focus on the role of a series of adipokines and discuss how they influence hepatic insulin sensitivity.

## 2. Adipokines and Hepatic Insulin Resistance

### 2.1. Adiponectin

Since the identification of adiponectin as a protein exclusively produced from adipocytes exclusively by Scherer et al. in 1995 [9], it has grabbed much attention by scientific communities, primarily due to its inverse relationship with hepatic insulin resistance. It was first named as adipocytes complement related protein of 30 kDa (ACRP30) on the basis of structural similarity with the complement C1q fraction [9]. At the same time other research groups also termed it as AdipoQ, adipose most abundant gene transcript 1 (apM1) or (gelatin-binding protein 28) GBP28, in both mice and humans [10]. Following the characterization of its genomic organization, comprising three exons and its 3q27 localization in 1999 [11] the protein was named adiponectin [12]. In 2004, the gene was named adipocytes C1q and collagen-domain-containing (ACDC), but actual nomenclature given by the Human Genome Organization (HUGO) nomenclature is now ADIPOQ [13].

The adipokine adiponectin is greatly expressed in human serum where it accounts for 0.01% of total plasma protein and is predominantly secreted by adipose tissue [14] (reports of secretion in differentiating preadipocytes [15] and placenta [16]) in inverse proportion to the body mass index [17]. A unique feature of the structure of adiponectin is its ability to assemble into several characteristic oligomeric isoforms, including trimers (low molecular weight (LMW)), hexamers (middle molecular weight (MMW)), and the oligomeric complexes comprising 18 protomers or above (high molecular weight (HMW)) [18]. Adiponectin presents predominantly in the circulation in the three oligomeric complexes cited previously [19]. Trimeric adiponectin is the basic unit block of adiponectin. Two LMW adiponectin molecules linked by disulfide bonds to form hexameric adiponectin [18]. Adiponectin may also form hetero-oligomers with additional members of the C1q/TNF-related protein (CTRP) family such as the CTRP9 [20].

The role of adiponectin in hepatic insulin resistance has evolved into a major factor and seen by many as therapeutic option now. The generalized effects of adiponectin on insulin resistance can be cited elsewhere [21, 22]. In this portion we will review the major studies describing the role of adiponectin and its receptors [23] in hepatic insulin resistance.

Hepatic insulin resistance is a hall mark for the spectrum of many diseases, and it is an independent predictor for metabolic disorders. Recent evidence suggests that visceral adipose tissue is a metabolic and inflammatory organ that signals and modulates the action and metabolism of the brain, liver, muscle, and cardiovascular system [24, 25]. The hormone plays a role in the attenuation of the hepatic metabolic disorders that may result in type 2 diabetes [26], insulin resistance, obesity [27], and nonalcoholic fatty liver disease (NAFLD) [28].

Recent data has exhibited more specification as most of the studies have implicated HMW oligomers in attenuation of hepatic insulin resistance. For instance, the synthesis of the HMW oligomers is necessary to mediate the insulin sensitizing effects of adiponectin on the suppression of hepatic gluconeogenesis in primary rat hepatocytes [29]. Moreover, HMW oligomers of adiponectin potently blunted hyperglycemia in diabetic mice through the inhibition of hepatic glucose production [30]. Further, acute injection of recombinant adiponectin enriched with the HMW oligomers resulted in a marked activation of AMP-activated kinase (AMPK) in the liver, while chronic infusion with this protein leads to prolonged alleviation of hyperglycemia and insulin resistance in *db/db *diabetic mice [31]. Animal studies are matched to the clinical data exhibiting that the ratio of HMW/total adiponectin correlates closely with hepatic insulin sensitivity [32]. The role of the HMW oligomer as a predominant active form of adiponectin mediating its hepatic actions is also supported by two recent independent reports demonstrating that the insulin-sensitizing effects of the peroxisome proliferator-activated receptor gamma (PPAR-*γ*) agonist thiazolidinediones were diminished in *ob/ob *obese mice with the targeted mutation of the adiponectin gene [33]. Notably, treatment with thiazolidinediones, which is an insulin sensitizer caused a selective increase of the HMW oligomeric adiponectin [32]. Pioglitazone, another insulin sensitizer, has remarkably increased the HMW adiponectin, which correlated greatly with the increased hepatic insulin action [34].

Plethora of animal data has been published describing the role of adiponectin in hepatic insulin resistance. Different animal models have supported this supposition widely. For example, a high scale study of adiponectin function in animal models of obesity, insulin resistance, and type 2 diabetes provided strong evidence that adiponectin promotes insulin sensitivity in muscle and in the liver [35]. Further, most of the adiponectin knockout mice were more insulin resistant than controls, although to different degrees, and this factor has been associated with hepatic insulin resistance [36].

Moreover, adiponectin-deficient mice showed mild insulin resistance in the liver with administration of standard diet [36]. Similar evidences were extracted on adiponectin receptor (AdipoR) studies, as adipoR1 and adipoR2 knockout mice exhibit mild insulin resistance [37]. Further, AdipoR2 knockout mice showed reduced diet-induced insulin resistance but promoted type 2 diabetes [38]. In another study, secretion of wild type adiponectin has been employed [39] and caused insulin sensitization when expressed in either liver [39] or adipose tissue [40]. Interestingly, infusion of adiponectin inhibited both the expression of hepatic gluconeogenic enzymes and the rate of endogenous glucose production by the liver. The latter was dependent on insulin, because adiponectin alone had no significant effect on glucose output in cultured hepatocytes. Obviously, both wild-type and type 2 diabetic mice were shown to reduce the expression of gluconeogenic enzymes and elevated phosphorylation of hepatic AMP-activated protein kinase (AMPK), and the effects were attributes to adiponectin due to its circulating levels [41]. Indeed AMPK has currently established itself as a target for adiponectin, and its phosphorylation has been linked with the adiponectin level [42]. It is worth mentioning here that most of the adiponectin effects on liver has been attributed to either AMPK activation or nuclear receptor involvement, and this activation is mediated by AdipoR2, but a recent ground breaking study refuted this as an only view and put forward new dimensions in future adiponectin research. Motoharu and his group have shown that adiponectin effects on hepatic system can be independent of AdipoR2 and can also be mediated by inflammatory cells [43].

Clinical studies have described the similar evidences regarding the role of adiponectin in liver insulin resistance. Instantly, adiponectin levels are associated, in healthy humans, with plasma concentrations of various liver function indices [44]. Further, a clinical study showed that plasma level of adiponectin was closely linked with hepatic lipids and insulin resistance in patients administered with pioglitazone [45]. Another clinical study supported the similar view and showed that increased adiponectin level could help restore the hepatic insulin resistance in severely obese women [46].

In conclusion, adiponectin is a promising target for hepatic insulin resistance and resultantly metabolic disorder, but further clinical research is required to translate similar impacts in humans keeping in mind the pleiotropic nature of this hormone.

### 2.2. Leptin

In 1994, ob gene was first cloned on mice chromosome 6 [47]. A year later, Halaas found that leptin was the gene product of ob in colon bacillus [48]. Leptin is a 16-KDa hormone secreted by adipocytes which plays a key role in the regulation of food intake, energy metabolism, saccharide, lipid metabolism, and so forth. The levels of leptin in adipose tissue and plasma are dependent on the amount of energy stored like fat as well as the status of energy balance. Therefore, leptin levels are higher in obese individuals and increase with overfeeding [49, 50].

Lots of studies reported that a strong positive correlation exists between the concentration of leptin and insulin sensitivity and obesity [51–54], but the anti-insulin resistance effect of leptin, independently of its activity on weight control, was also reported [55, 56]. The mechanisms that explain the direct role of leptin in hepatic insulin resistance are not well understood. Studies in animal model showed that exogenous leptin administration leads to a dramatic improvement in insulin resistance that is independent of decreased caloric intake [57]. Clinical studies showed that chronic leptin treatment ameliorates insulin-stimulated hepatic and peripheral glucose metabolism in severely insulin-resistant lipodystrophic patients [58]. A study showed that in leptin receptor-deficient Koletsky rats, adenovirally induced expression of leptin receptors in the area of the hypothalamic arcuate nucleus improved peripheral insulin sensitivity via enhancement of suppression of hepatic glucose production, with no change of insulin-stimulated glucose uptake or disposal, and leptin regulated hepatic insulin sensitivity via phosphatidylinositol-3-OH kinase (PI3K) signaling in the liver of this animal model [59].

In summary, leptin acts as an important target to improve the hepatic sensitive to insulin, and the precise mechanisms about leptin underlying hepatic insulin resistance should be investigated by experiments *in vivo* and *in vitro*.

### 2.3. Resistin

Resistin is a member of a family of cysteine-rich proteins referred to as resistin-like molecules, termed RELMs, which include RELM-*α*, RELM-*β*, and the recently discovered RELM-*γ* [60]. It was discovered in 2001 by Dr Mitchell A. Lazar [61] and was called resistin, since mice injected with resistin exhibited insulin resistance. Crystal-lographic studies of resistin have determined its complex hexameric structure. Resistin is proved to circulate in two distinct assembly states, likely corresponding to hexamers and trimers. Infusion of an intertrimer disulfide bonds-lacking resistin showed more potent effects on hepatic insulin sensitivity than those observed with wild-type resistin [62]. This result suggested an activation effect of different intertrimer disulfide bonds and further suggested a potential target site for the receptor interaction.

In rodents, resistin primarily expressed in and secreted from mature adipocytes, with some expression in pancreatic islets and portions of the pituitary and hypothalamus. However, resistin is expressed primarily by macrophages and seems to be involved in the recruitment of other immune cells and the secretion of proinflammatory factors in humans [63]. The diverse expression and regulatory patterns may be explained by the evidence that human and mouse resistins have diverse genomic organizations [64]. Moreover, at the protein level, human resistin is only 55% identical to its murine counterpart.

A study in rodents showed that the infusion of resistin rapidly induced severe hepatic insulin resistance, resulting in a reduced insulin-mediated suppression of gluconeogenesis and increased glycogenolysis [65]. Consistent with these reports, resistin induced insulin resistance with a robust decrease in insulin-stimulated phosphorylation of Akt and glycogen synthase kinase3 (GSK3) human liver cell line HepG2 cells [66], pointing to a specific role of resistin in the initiation of hepatic insulin resistance. Furthermore, mice treated with an antiresistin IgG [67] or expressing a dominant negative resistin [68] exhibit low fasted blood glucose levels due to reduced hepatic glucose production. 

In a word, resistin could induce hepatic insulin resistance by inhibiting the phosphorylation of Akt and GSK3, and its effect on insulin sensitivity is opposite to those reported for the adipocyte-secreted hormone adiponectin, which increases insulin sensitivity of the same liver-specific functions.

### 2.4. IL-6

IL-6 is secreted by many cell types, including immune cells, fibroblasts, endothelial cells, myocytes, and a variety of endocrine cells [69]. Adipose tissue contributes to 10–35% of circulating IL-6 [70] in resting, healthy humans, and the production is greater in obese subjects [71]. It is a multifunctional cytokine that has been well known for its anti-inflammatory and proinflammatory effect in immune responses. IL-6 signals through a cell-surface type I cytokine receptor complex consisting of the specific receptor subunit IL-6 receptor (IL-6R) and the signal-transducing component glycoprotein 130 (gp130), which is the common signal transducer for several cytokines including leukemia inhibitory factor (LIF), ciliary neurotropic factor, oncostatin M, IL-11, and cardiotrophin-1 [72]. In contrast, the expression of IL-6R is restricted to certain tissues. IL-6 interacts with its receptor IL-6R and gp130 protein to form a complex that activates the receptor IL-6R.

The inflammatory regulator IL-6 has also emerged as a factor that is implicated in hepatic insulin resistance. However, the role of IL-6 in the etiology of insulin resistance is not fully understood. Impaired insulin receptor signaling and insulin-dependent glycogen synthesis were found in HepG2 cells and primary mouse hepatocytes acutely pretreated with IL-6 [73]. IL-6 caused reduced insulin signal transduction in the liver of mice [74]. These results seemed to be consistent with the hypothesis that IL-6 may have a negative effect on insulin resistance. But in healthy humans under the basal condition, treatment by recombinant human IL-6 (rhIL-6) at a physiological concentration neither impaired the whole body glucose disposal nor increased endogenous glucose production [75]. In patients with type 2 diabetes, splanchnic glucose output did not increase with acute infusion of rhIL-6 [76], while glucose disposal was not impaired, suggesting that IL-6 might have favorable action on insulin action.

IL-6 mediates insulin sensitivity through many distinct mechanisms. Of note, there is a significant correlation between the level of IL-6 in adipose tissue and several metabolic parameters such as fasting plasma glucose, basal and insulin-stimulated glucose transport, and whole-body glucose disposal [77]. Thus, IL-6 may play an important role in insulin action in these subjects. Moreover, IL-6 mediated suppressor of cytokine signaling (SOCS-3) pathway in liver, leading to impairment of insulin actions [78].

To sum up, the effect of IL-6 on hepatic insulin sensitivity is uncertain; it appeared to be determined by whether it is present acutely or chronically; the latter is the setting associated with insulin resistance.

### 2.5. TNF-*α*

TNF-*α* is a proinflammatory cytokine produced by various types of cells, mainly but not only inflammatory cells like macrophages and lymphocytes. The release of TNF-*α* in noninflammatory cells has also been reported, at lesser degrees. Since the first recognized sequencing of TNF-*α* in 1984, it has established itself as a prime proinflammatory cytokine [79], making it the subject of continuous pondering by researchers. It is now clear that TNF-*α* is involved in array of pathological conditions like obesity, congestive heart failure, inflammations, and insulin resistance [80], nevertheless the exact role and degree of its effects are still not clearly understood.

TNF-*α* has been proposed as a link between adiposity and the development of insulin resistance [81], because the majority of type 2 diabetic subjects are obese [82]. Elevated levels of TNF-*α* have been observed in obese and insulin resistance humans and animals [83]. TNF-*α*-mediated pathway interference has shown protection in diet induced animal models of obesity and metabolic syndrome [84]. A clinical study has observed improvement in the insulin sensitivity under anti-TNF-*α* therapy [85]. Anti-TNF-*α* therapy has also shown to attenuate in insulin resistance in animal studies involving fructose-red rats [86]. Further, TNF-*α* expression is increased in adipose tissue in obese rodents and humans [87], and reducing TNF-*α* signaling either by knocking TNF-*α* out or by infusing blocking antibodies can reduce insulin resistance in obese rodents [88]. More recent clinical evidence confirms the proportional rise of TNF-*α* and IR with parallel increase in body mass index (BMI) factor [89]. A recent cohort study on nondiabetic patients with insulin resistance has shown concomitant decrease in TNF-*α* level accompanied by an improvement in insulin resistance after administration of rosiglitazone [90]. Another reported study has observed the direct dose-dependant effects of TNF-*α* and higher doses linked directly to insulin sensitivity [91]. Moreover, insulin stimulating effects on glycemic levels and glucose uptake have been reversed by the administration of TNF-*α* [92]. A clinical trial which consists of 15 women with normal glucose tolerance and developed gestational diabetes mellitus has proved TNF-*α* as the sole biomarker and predictor for insulin resistance during pregnancy [93]. In one rodent model, obese mice lacking either TNF-*α* or its receptors showed protection against developing insulin resistance [94]. Liver resident macrophages (Kupffer cells) in human body are the primary source of TNF-*α*; diet induced macrophages which subsequently release the TNF-*α* are found to be involved in insulin resistance in paracrine signaling.

TNF-*α* is reported to demonstrate wide range of effects in pathogenesis of insulin resistance. The precise signaling pathway is, however, not clear yet even after scores of studies and scientific data put forward on this particular subject. However, the most attention grabbing signaling pathway in liver supported by numerous evidences is through the phosphorylation of the insulin receptor substrate-1 (IRS-1) protein on serine residues. This could prevent its interaction with the insulin receptor beta subunit and stop the insulin signaling pathway [95–97]. However, the precise role of TNF-*α* in human insulin signaling requires extensive scientific investigation in future to clear its signaling pathway.

Conversely, numerous clinical studies have not been able to support similar findings and failed to prove its role in the relationship between type 2 diabetes coexisting with insulin resistance [98, 99]. TNF-*α* antibody infusion in humans was shown not to alter insulin sensitivity, fueling lingering uncertainty about the biological relevance of this pathway in human insulin resistance states [100].

Conclusively, TNF-*α* role in modulating insulin resistance with no doubt varies from species to species and depends on animal models adopted for particular study. Further, due to its release by various types of cell types involving both inflammatory and noninflammatory cells which then can be involved in insulin resistance pathogenesis both by autocrine or paracrine manner, TNF-*α* is the subject of uncertainty regarding its precise role in insulin resistance. Without doubt, further scientific work is required in order to unravel its exactness in insulin resistance.

## 3. Summary

**Figure 1:**
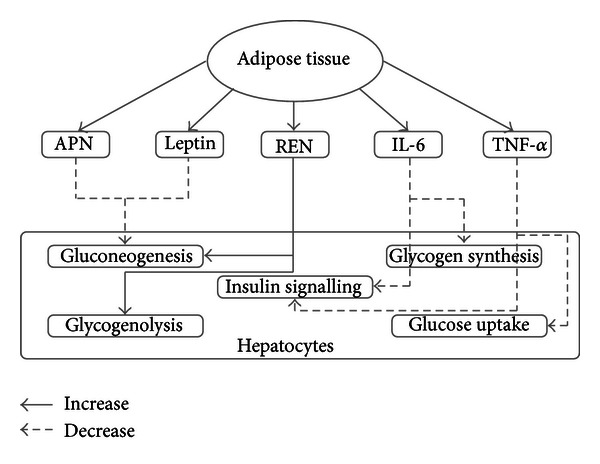
The effects of adipokines on hepatic glucose metabolism and insulin signalling. Both of adiponectin (APN) and leptin can decrease hepatic gluconeogenesis. Resistin (REN) can increase hepatic gluconeogenesis and glycogenolysis. Moreover, interleukin-6 (IL-6) can decrease glycogen synthesis, and tumor necrosis factor *α* (TNF-*α*) can decrease glucose uptake in liver. Both of them can block hepatic insulin signalling by interfection of insulin receptor signalling and insulin signal transduction.

During the last couple of years it has been shown that adipokines play an important role in physiology and pathophysiology of insulin sensitivity. Our review summarizes recent findings regarding the relationship between adipocytokines and hepatic insulin resistance (Figure 1). The mechanisms by which adipocytokines promote or relieve insulin resistance are complex, and our understanding is yet incomplete. According to most of the recent studies, it seems that excessive adipose tissue may be detrimental partially through secretion of the following cytokines: TNF-*α*, IL-6, and resistin. In contrast, the presence of adipose tissue is vital in the prevention of hepatic insulin resistance, at least in part, via secretion of the following cytokines: leptin and adiponectin. Indeed, adipocytokines are major regulators of hepatic insulin sensitivity potentially linking insulin resistance and obesity. Further work is needed to clearly determine how they regulate the insulin signaling in hepatocytes and influence insulin sensitivity in other tissues (e.g., muscle and adipose tissue) in rodents and human and their contribution to glucose homeostasis in diabetes.
